# Technological Advances to Address the Challenging Abdominal Aortic Aneurysm Neck

**DOI:** 10.31083/j.rcm2403070

**Published:** 2023-02-24

**Authors:** Justin M George, Christopher M Hatzis, Krystina N Choinski, Rami O Tadros, Peter L Faries, Michael L Marin

**Affiliations:** ^1^Division of Vascular Surgery, Department of Surgery, The Icahn School of Medicine at Mount Sinai, New York, NY 10029, USA

**Keywords:** aortic aneurysm, evar, neck, endoleak, endoanchor, fenestrated, branched

## Abstract

There have been significant technologic advances in endovascular aortic therapies since the introduction of conventional infrarenal endovascular aortic aneurysm repair (EVAR). These advances have sought to address the weaknesses of conventional EVAR- particularly the difficult or “hostile” infrarenal aortic aneurysm neck. We review anatomical features that create a hostile neck and the most recent advancements to overcome these limitations. EndoAnchors replicate open suture fixation to seal endograft to aortic tissue and have been shown to be useful as a prophylactic measure in short, angulated necks as well as therapeutic for type Ia endoleaks. Fenestrated EVAR (FEVAR) devices such as the Z-fen (Cook Medical, Bloomington, IN, USA) raises the seal zone to the suprarenal segment while maintaining renal perfusion. Finally, multibranch aortic grafts such as the Thoracoabdominal Branch Endoprosthesis (Tambe; W. L. Gore & Associates, Flagstaff, AZ, USA) raise the seal zone above the visceral segment and can be used off the shelf with promising results.

## 1. Introduction

The introduction of endovascular aortic aneurysm repair (EVAR) in 1991 revolutionized aortic therapy and has rapidly become the first line treatment modality for anatomically suitable abdominal aortic aneurysms (AAA) [1, 2]. Despite EVAR becoming widely propagated and comfort with endovascular techniques rapidly advancing, nearly 40% of patients have complex anatomy unsuitable for conventional EVAR [3, 4]. Proximal aneurysm neck anatomy is the most important anatomical feature with a “hostile” neck significantly increasing the risk of type Ia endoleak and aneurysm related mortality after EVAR [5].

Commonly cited aortic neck characteristics that create a hostile neck include length shorter than 15 mm, large diameter, tapered/reverse tapered anatomy, mural thrombus, circumferential calcification, and angulation [6]. Currently available EVAR devices have proximal diameters of 22 mm to 36 mm with an instruction-for-use (IFU) to seal within aortic neck diameters 18 mm to 32 mm. Early solutions to large diameter necks were simply larger diameter grafts up to 36 mm in size; however, follow up data demonstrated significantly increased rates of proximal fixation failure [7]. Conventional EVAR use in aortic neck diameters ≥28 mm has been associated with increased neck-related adverse effects including type Ia endoleak and rupture [7, 8, 9].

With respect to neck length, the first generation of EVAR devices had an IFU neck length requirement of 15 mm. Standard EVAR in infrarenal neck lengths <10 mm has been associated with higher type Ia endoleaks [10]. Furthermore, neck angulation can also compromise proximal seal. Most conventional EVAR devices require neck angulation <60 degrees to allow adequate opposition of the device to the aortic wall. Many studies have demonstrated that high degrees of neck angulation are associated with EVAR failure [10, 11]. This failure of adequate endograft-aortic wall opposition also occurs in conical and reverse tapered neck configurations.

Multiple technologic advances have occurred to address issues with anatomic constraints. In this review, we will discuss advances to attack the challenge of complex, hostile abdominal aortic aneurysm necks. Each of these technologies has advantages and contraindications that will be discussed. All play a role in managing the hostile abdominal aortic neck and should be selected based on patient profile and anatomy.

## 2. Discussion

### 2.1 EndoAnchors

EndoAnchors are a catheter based fixation system designed to mimic open interrupted suture fixation and affords the ability to achieve significantly higher degree of fixation particularly over a very short longitudinal distance of aortic tissue [12]. Traditional EVAR fixation involves use of radial force and barbs; however, EndoAnchor technology attempts to replicate open suture fixation. There have been several EndoAnchor and endosuture devices that began investigational use since 2008 [13]. The Aptus Heli-FX EndoAnchor system (Medtronic Vascular, Santa Rosa, CA, USA) is currently the only FDA approved device for EndoAnchor fixation. The system is designed to penetrate both the endograft fabric and aortic tissue in order to seal the device in the infrarenal neck [14].

EndoAnchors can be used either prophylactically in hostile necks to prevent endograft migration, or to treat type Ia endoleaks after endograft proximal fixation failure [14, 15, 16]. Placement of EndoAnchors has been shown to decrease the rate of aortic neck dilation thereby exerting a protective effect on endograft seal zone and preventing device migration [17].

**Fig. 1.**
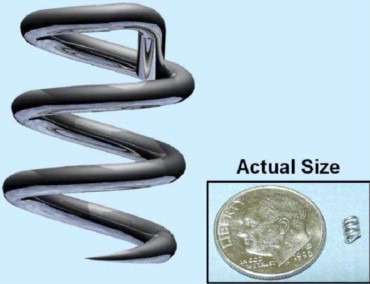
**Heli-FX EndoAnchor Implant**. The Heli-FX EndoAnchor implants are 0.5 mm thick with a tapered end designed to replicate a “SH” surgical needle. They are 4.5 mm in length and when correctly deployed, penetrate the endograft fabric and aortic wall, thus fixating the endograft in place.

The Heli-FX EndoAnchor is a helical 0.5 mm thick metallic alloy with 4.5 mm length and a tip tapered to replicate an “SH” needle (Fig. 1) [12].

**Fig. 2.**
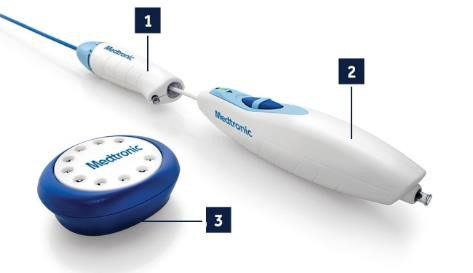
**Heli-FX EndoAnchor Components**. Heli-FX EndoAnchor Components include the steerable delivery Heli-FX guide sheath (1), the motorized Heli-FX Applier (2), and the EndoAnchor cassette containing 10 EndoAnchor implants (3).

The EndoAnchor is loaded into the Heli-FX Applier device using a motorized, automated system (Fig. 2). A 16 French steerable sheath is then angled perpendicular to the endograft in order to gain full opposition to the wall. The applier is placed through the steerable sheath and the EndoAnchor is deployed half way with the option to retrieve and reposition. After obtaining adequate position, the EndoAnchor can be fully deployed.

Subgroup analysis of the ANCHOR (Aneurysm Treatment Using the Heli-FX EndoAnchor System) registry has shown promising outcomes with type Ia endoleak rates of only 1.5% in patients with a challenging proximal neck [15]. Graft migration in the primary EndoAnchored EVAR group was 2.0% [18]. Aneurysm sac regression has also been shown to be significantly higher in patients treated with EndoAnchors than those without [19]. Complications of EndoAnchors are rare and primarily limited to maldeployment; however, meta-analysis have demonstrated technical success rates as high as 97% [20]. Relative contraindications for Heli-FX EndoAnchors include infrarenal neck length <8 mm, neck diameter >34 mm, neck angulation >90°, circumferential neck calcification, and circumferential aortic mural pathology >2 mm in thickness [21]. Circumferential calcium may prevent penetration of the EndoAnchor into the aortic wall and thus adequate fixation.

#### Case 1: Primary EndoAnchor use in hostile infrarenal neck

**Fig. 3.**
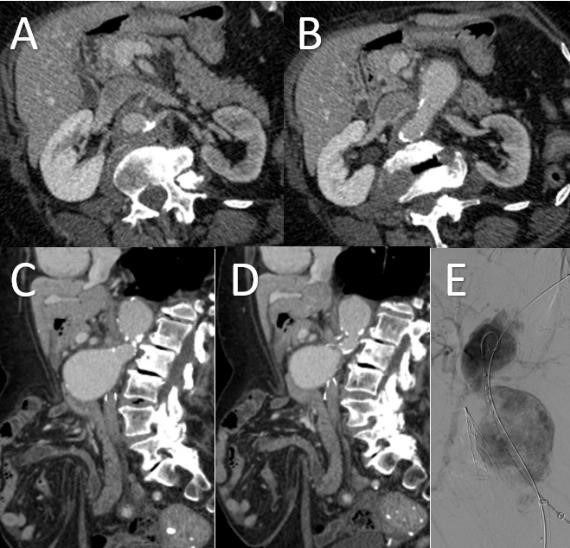
**Preoperative Imaging of Infrarenal Aortic Aneurysm with Hostile Neck**. Preoperative axial (A,B), sagittal (C,D), and angiographic (E) images of a 6.3 cm infrarenal abdominal aortic aneurysm with short, angulated, conical neck. Conventional EVAR would likely fail given the hostile aortic aneurysm neck.

**Fig. 4.**
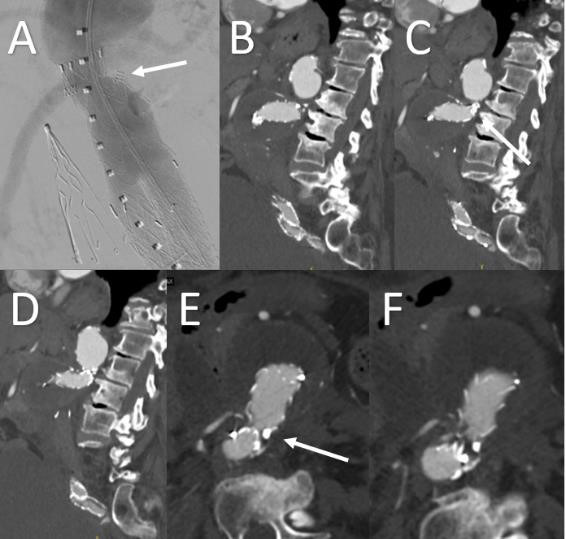
**Postoperative and Follow-up Imaging of Infrarenal Aortic Aneurysm with Hostile Neck Managed with EVAR and adjunctive Heli-FX EndoAnchors**. Completion aortogram (A) and follow up sagittal (B,C,D) and axial (E,F) images after repair of infrarenal abdominal aortic aneurysm using traditional EVAR device in conjunction with Heli-FX EndoAnchors (white arrow). The EndoAnchors can be seen penetrating the endograft fabric into the aortic tissue.

An 86-year-old female with extensive medical history who was prohibitive risk for open surgical intervention presented with 6.3 cm infrarenal abdominal aortic aneurysm with a short, highly angulated, conical shaped neck (Fig. 3). A Gore Excluder (W. L. Gore & Associates, Flagstaff, AZ, USA) was used in combination with Heli-FX EndoAnchors to ensure proximal seal. Intraoperative completion aortogram and follow-up CT demonstrated excellent seal with no evidence of device migration or endoleak (Fig. 4). The patient has undergone two years of follow up with no evidence of endoleak or aneurysm sac enlargement.

#### Case 2: EndoAnchors for Treatment of Type Ia Endoleak

**Fig. 5.**
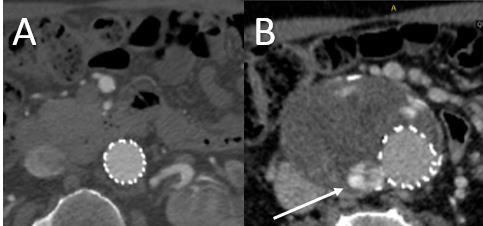
**CT after prior conventional EVAR for AAA demonstrating type Ia endoleak (white arrow)**. The infrarenal neck is dilated and short (A). This compromised proximal fixation of the Endograft and resulted in type Ia endoleak (B).

**Fig. 6.**
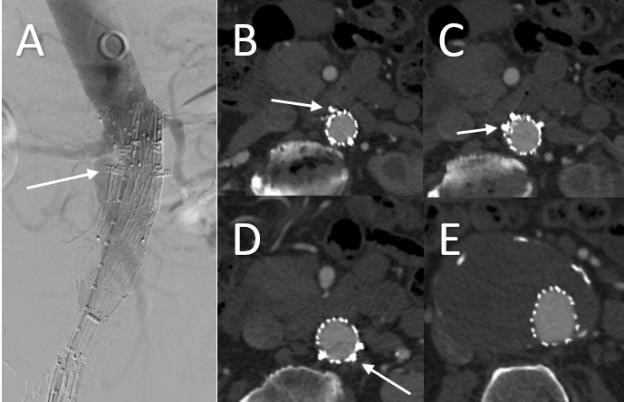
** Type Ia Endoleak after previous EVAR managed successfully with Aortic Cuff and Heli-FX EndoAnchors**. Completion Aortogram (A) and follow up CT (B,C,D,E) demonstrating resolution of endoleak with use of proximal aortic cuff and Heli-FX EndoAnchors (white arrow).

A 78-year-old male with history of infrarenal AAA s/p EVAR with Cook Zenith Flex (Cook Medical, Bloomington, IN, USA) presented with type Ia endoleak and enlargement of aneurysm sac to 7.8 cm on 2 year follow up CT and intraoperative angiogram (Fig. 5). The patient had proximal aortic cuff placement with a 25 mm × 25 mm × 49 mm Medtronic Endurant Aortic Cuff (Medtronic Vascular, Santa Rosa, CA, USA) and Heli-FX EndoAnchors (Medtronic Vascular, Santa Rosa, CA, USA) with resolution of endoleak (Fig. 6). The patient has since been followed with serial CT scans for 2 years with aneurysm sac regression and no evidence of endoleak.

### 2.2 Fenestrated Devices

The first reported cases of juxtarenal AAA treated with fenestrated EVAR (FEVAR) were published in 1999 [22, 23]. In the interim, aided by the first FDA approval of a fenestrated device in 2012, FEVAR use has become more prevalent in the general population. The FEVAR technique was developed as a solution for treatment of AAAs with unsuitable, short proximal aortic necks [24, 25]. Prior studies have demonstrated a higher risk of complications when addressing these short-neck (<15 mm) or pararenal aneurysms with either conventional infrarenal EVAR [26] or with open repair [27, 28]. Basic science models have demonstrated that the rate of dilation differed in different segments of the aorta with the highest rates at the level of the lowest renal and the lowest rates of dilation at the level of the mesenteric vessels [29]. The fenestrated technique allows for proximal extension of the seal zone to a more stable area within the suprarenal aortic neck.

While there are multiple fenestrated devices in the pathway from development to regulatory approval, they all share certain characteristics and nomenclature [30]. The ‘standard’ fenestrated endograft is defined by two, small, rounded fenestrations and one semicircular scallop. The two fenestrations allow perfusion to renal artery branches off the aorta which would otherwise be covered by endograft fabric. Fenestrations are made to vary in diameter dependent on patient anatomy and are typically used as conduits for renal artery stenting. The scallop is a semicircular opening which incorporates the proximal edge of the FEVAR endograft and typically allows filling of the superior mesenteric artery. Modern fenestrated endografts are composite endografts with fenestrations arising from a proximal tube graft. The proximal tube graft segment allows temporary endograft rotation during cannulation of renal arteries without compromising graft position. A bifurcated endograft is subsequently deployed within the distal end of the tube graft. FEVAR deployment is completed with placement of distal iliac limbs.

Certain anatomic criteria must be taken into account when planning fenestrated endovascular aortic repair. In cases with hostile aortic aneurysm necks, the choice of proximal seal zone is of the utmost importance. Some of the general rules of infrarenal EVAR also apply to this more complex subset of aortic aneurysms. In particular, proximal seal should be obtained in a relatively straight segment of aorta, with parallel walls, and without the presence of significant thrombus or calcification. However, in contrast to infrarenal EVARs where the desired seal length is ~15 mm, in FEVAR the infrarenal neck length requirement is only 4mm which produces a target sealing zone length is 20–40 mm [31]. This extended seal zone length is made possible by the ability to proximalize the seal zone into the suprarenal aortic segment via fenestrations.

**Fig. 7.**
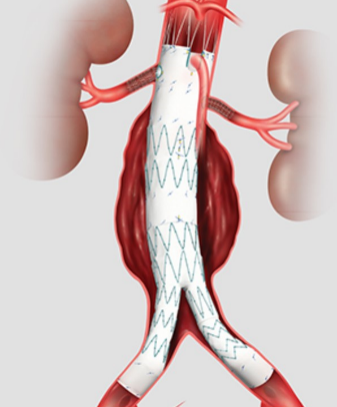
**Schematic of Fenestrated Endovascular Device**. The only FDA approved Fenestrated endovascular aortic repair (FEVAR) device is the Z-fen (Cook Medical, Bloomington, IN, USA) and is custom made to fit patient anatomy with up to two renal fenestrations and one SMA scallop. It can be used in difficult aortic necks as it extends the seal zone into the renal segment.

There is currently one FDA approved fenestrated endograft for treatment of short-neck infrarenal and juxtarenal AAA - the Zenith Fenestrated (ZFen) Endovascular Graft (Cook Medical, Bloomington, IN, USA) (Fig. 7). The approved anatomic instructions-for-use of this device include: Proximal aortic neck length ≥4 mm and <15 mm, proximal aortic neck diameter <31 mm, proximal aortic neck angle <45°, and non-aneurysmal common iliac arteries (<21 mm) [32].

Use of the ZFen device ‘Off-IFU’ for the treatment of pararenal, suprarenal, and thoracoabdominal aortic aneurysms has also been described [33, 34]. There are a few factors that are crucial to optimize successful treatment of these more complex aneurysms with fenestrated endografts. First, it is necessary to place covered (as opposed to bare metal) stent grafts in the renal arteries. Second, the renal artery stents must be placed with adequate apposition to the endograft main body fenestrations, preventing dissociation of the two. Third, proper wall apposition of the endograft main body to the native aortic neck must be obtained. Proper wall apposition prevents movement of the endograft relative to the native aorta, decreases the likelihood of endoleak, and prevents renal artery stent migration either away from the endograft main body or from the renal artery ostia [35]. A recent systematic review of target vessel stent grafts during fenestrated and branched EVAR demonstrated higher complication rates in renal artery stent grafts compared to visceral artery stent grafts (6% vs 2%), similar re-intervention rates, and similar complication profiles for self-expanding versus balloon expandable stent grafts [36]. While fenestrated devices have shown good efficacy for the treatment of pararenal AAAs, the development of thoracoabdominal and branched endografts that are specifically designed for this patient population continues to enhance treatment options for these complex pathologies. Furthermore, fenestrated devices are custom made to fit patient anatomy thus requiring up to 6 weeks for device construction and delivery. The delay in availability restricts use in emergent and urgent cases.

#### Case 3: FEVAR for Treatment of 6 cm Juxtarenal AAA

**Fig. 8.**
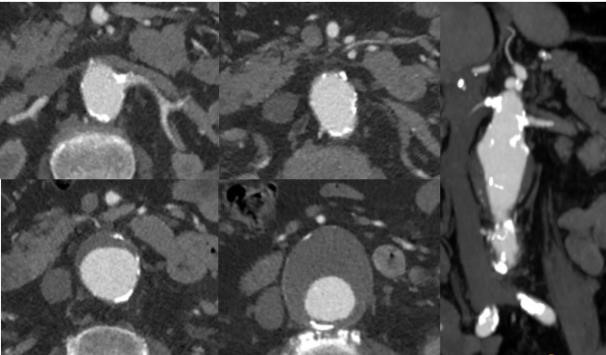
**Preoperative Imaging of Abdominal Aortic Aneurysm with Hostile Neck**. Preoperative CTA demonstrating 6.3 cm AAA with short, conical neck making conventional infrarenal EVAR challenging.

**Fig. 9.**
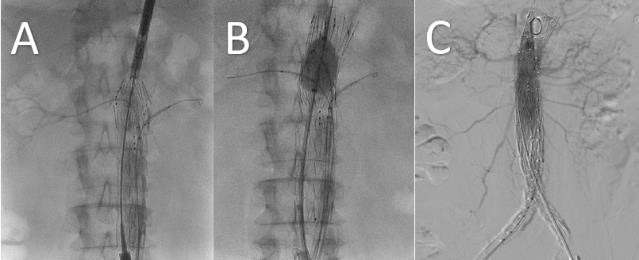
**Fenestrated Endovascular Aortic Repair**. Intraoperative images demonstrating positioning of custom made fenestrated aortic device with partial deployment allowing cannulation of bilateral renal arteries (A), deployment of top cap and proximal ballooning to ensure proximal aortic wall apposition (B), and completion aortogram demonstrating successful exclusion of aortic aneurysm with filling of bilateral renal artery stent grafts (C).

**Fig. 10.**
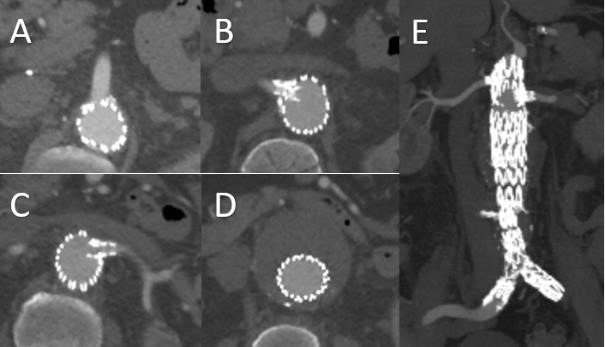
**Follow up Imaging after Successful Management of Abdominal Aortic Aneurysm with Hostile Neck Using FEVAR**. Follow up CT after FEVAR for 6.3 cm aneurysm with conical neck demonstrating SMA scallop (A), bilateral renal artery stents (B,C), with successful exclusion of aneurysm sac (D,E).

An 82-year-old male with hypertension, hyperlipidemia, and diabetes mellitus was found to have a 6.3 cm AAA with short conical neck (Fig. 8). Conventional infrarenal EVAR was not suitable given neck anatomy. A custom fenestrated device was partially deployed allowing cannulation of bilateral renal arteries (Fig. 9A). After successful cannulation of bilateral renal arteries, and confirmatory cannulation of superior mesenteric artery, the proximal top cap was released and balloon angioplasty of proximal graft confirmed sufficient aortic wall apposition prior to renal artery stent deployment with 6 mm Viabahn balloon-expandable (VBX) stent grafts (W. L. Gore & Associates, Flagstaff, AZ, USA) (Fig. 9B). Completion aortogram after bilateral renal artery stent graft placement through the fenestrations demonstrated successful aortic aneurysm exclusion, as well as perfusion to bilateral renal arteries and to the superior mesenteric artery (Fig. 9C). Follow up CT after FEVAR demonstrated patency of SMA, bilateral renal stent grafts, with successful exclusion of the aneurysm sac (Fig. 10). The patient has completed three years of follow up with no evidence of endoleak or aneurysm sac enlargement. This case illustrates management of hostile infrarenal neck by extending the neck proximally using fenestrated technology.

### 2.3 Thoracoabdominal Devices

Current research has focused on expansion of endovascular treatment of complex pararenal and thoracoabdominal aortic aneurysms with off-the-shelf multibranched aortic stent grafts. These devices have the advantage of avoiding the treatment delay encountered for custom-made aortic devices [37]. Off-the-shelf investigational multibranched aortic devices include the Gore Excluder thoracoabdominal branch endoprosthesis (TAMBE; W. L. Gore & Associates, Flagstaff, AZ, USA), the E-nside multibranch stent graft system (Jotec GmbH, Hechingen, Germany), and the Zenith t-Branch (Cook Medical, Bloomington, IN, USA) [38, 39, 40, 41, 42]. The Zenith t-Branch is not available in the United States and it’s use is currently being investigated in Europe [43]. The focus of this review article will be on the design and applications of the TAMBE stent graft for aortic aneurysms. The multi-branched aortic graft allows for endovascular treatment of AAA with hostile necks by raising the seal zone above the renovisceral segment to healthy aortic tissue.

The GORE Thoracoabdominal Branch Endoprosthesis (TAMBE) stent graft is an investigational aortic graft for endovascular repair of complex abdominal aortic aneurysms with incorporation of renal and splanchnic arteries. It is based on the GORE Excluder AAA (W. L. Gore & Associates, Flagstaff, AZ, USA) platform using a nitinol stent frame and conformable expanded polytetrafluoroethylene technology [39]. It is currently being investigated in multiple trials for patients with complex aortic pathology, including type IV thoracoabdominal aortic aneurysms, juxtarenal, and pararenal aneurysms [44, 45]. It is an off-the-shelf and multicomponent system composed of a multibranch stent graft, distal bifurcated component, and iliac limb extensions [40]. Target vessel stenting is performed using Gore Viabahn balloon-expandable (VBX) stent grafts (W. L. Gore & Associates, Flagstaff, AZ, USA).

The TAMBE features 4 portals for renal, celiac, and SMA stents. There is antegrade or retrograde orientation for the renal artery portals, accessed via brachial-axillary or femoral artery approach [40]. The antegrade renal portal device features a proximal diameter of 31 or 37 mm, 160 mm length, and 20 mm distal diameter. The retrograde renal portal device has a proximal diameter of 26, 31, or 37 mm, 215 mm length, and distal diameter of 20 mm. The device requires a 22 Fr introducer sheath, except for the 31 mm antegrade configuration, which requires a 20 Fr sheath. Additionally, for antegrade access a 12 Fr sheath for the brachial or axillary artery is utilized. The TAMBE device features preloaded removable guidewire tubes introduced through each portal to facilitate cannulation of visceral vessels with either 0.014 or 0.018 guidewires [40].

The TAMBE aortic components require a proximal aortic neck from 22 to 34 mm in diameter to achieve adequate sealing [39]. The TAMBE graft can be deployed alone or in combination with a proximal thoracic stent graft, the GORE Conformable TAG Thoracic Endoprosthesis (CTAG; W. L. Gore & Associates, Flagstaff, AZ, USA). The CTAG requires an thoracic aortic diameter of 19.5 to 32 mm. Aortic aneurysms extending up to 65 mm proximal to the origin of the celiac trunk can be treated without the use of a proximal thoracic graft. For aneurysms greater than 65 mm above the celiac trunk, the thoracic stent graft is deployed prior to the TAMBE aortic stent graft. Additional aortic requirements are a proximal seal zone of at least 20 mm, aortic neck angle less than 60 degrees at the proximal seal zone, and inner aortic diameter of 20 mm or greater at the level of the visceral vessel origin [39]. Iliac artery diameter can range from 8 to 25 mm with at least a distal seal zone.

TAMBE requires femoral access vessels at least 8.2 mm in diameter and one brachial or axillary artery access site with a minimum of 4.7 mm diameter. There can be no more than 4 renovisceral target vessels. Renal artery diameters that can be treated range from 4 to 10 mm, celiac and superior mesenteric artery diameters that can be treated range from 5 to 12 mm, and the length of each visceral vessel landing zone must be at least 15 mm. The celiac and SMA portal outlets are oriented from 10 to 30 mm above the celiac trunk and the distance from the celiac trunk to the aortic bifurcations must be 95 mm or greater [39].

TAMBE allows endovascular treatment of abdominal aortic aneurysms with hostile necks by raising the seal zone to the visceral segment while maintaining renovisceral perfusion through branch grafts. Many “hostile” necks are in reality juxta/pararenal aneurysms, which we now have the technology to treat with multi-branched aortic stent graft technology [39]. There has been excellent technical success and good short-term outcomes in early investigations of the TAMBE device [40]. For 13 patients with a pararenal or extent IV thoracoabdominal aortic aneurysms, the technical success rate for TAMBE deployment was 92% without any mortalities, aneurysm ruptures, or conversion to open surgery. Morbidity was low, with a mean hospital stay of 5 days and four patients with adverse events secondary to intra-operative blood loss [40]. One patient required a secondary procedure for type Ia endoleak at the renal stent. 30 day post operative imaging revealed patent target vessels and no type I or III endoleak in all patients [40].

#### Case 4: TAMBE for Treatment of a Juxtarenal Aortic Aneurysm

**Fig. 11.**
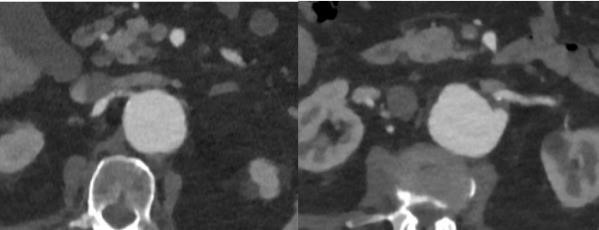
**Preoperative CT demonstrating a juxtarenal 5.5 cm aneurysm with no infrarenal neck necessitating treatment with a branched endograft system**. Conventional EVAR is not possible given the aorta measures approximately 35 mm at the level of the renal arteries.

**Fig. 12.**
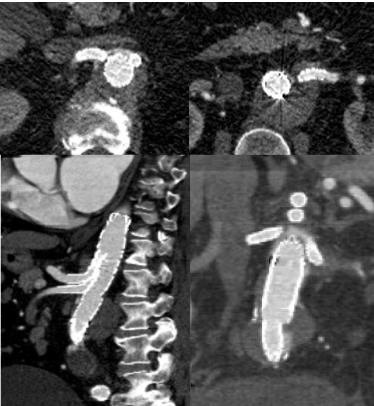
**Follow up CT demonstrating successful treatment of juxtarenal 5.5 cm aneurysm with an off the shelf multibranch endograft (TAMBE). **Both renal arteries, the celiac artery, and SMA and covered stent grafts into the main aortic body device allowing the seal zone to be raised well above the renovisceral segment.

An 83-year-old male with history of atrial fibrillation, diabetes, hypertension, and coronary artery disease was found to have an enlarging 5.5 cm juxtarenal aortic aneurysm. Preoperative CT demonstrated the infrarenal aorta was 5.0 cm in size and not suitable for any conventional infrarenal EVAR device (Fig. 11), thus he was enrolled in the TAMBE trial. The device was deployed above the celiac artery in healthy aortic tissue and all 4 renovisceral vessels were cannulated from axillary access and stented with VBX stent grafts. Follow up CT demonstrated successful exclusion of the aneurysm with patency of all renovisceral target vessels (Fig. 12). The patient has completed two year follow up imaging which demonstrated no evidence of endoleak or aneurysm sac enlargement with patency of all target vessel stent grafts. This complex abdominal aortic aneurysm could not have been treated using conventional EVAR technology; rather, was successfully treated by raising the seal zone above the visceral segment using the multibranch TAMBE device.

## 3. Conclusions

The compromised, hostile aortic neck has been shown to increase the risk of proximal seal failure in standard EVAR. Advances in endograft technology have addressed this with options to seal in shorter necks with EndoAnchors, raise the seal zone to the suprarenal segment using FEVAR, or raise the seal zone to the visceral segment with branched devices such as TAMBE. These newer devices show promising results in the treatment of complex aortic pathology. As with all aortic intervention, treatment must be customized based on patient profile and anatomy.
